# Subcortical cytoskeleton periodicity throughout the nervous system

**DOI:** 10.1038/srep22741

**Published:** 2016-03-07

**Authors:** Elisa D’Este, Dirk Kamin, Caroline Velte, Fabian Göttfert, Mikael Simons, Stefan W. Hell

**Affiliations:** 1 Department of NanoBiophotonics, Max Planck Institute for Biophysical Chemistry, Am Fassberg 11, 37077 Göttingen, Germany; 2 Department of Cellular Neuroscience, Max Planck Institute of Experimental Medicine, Hermann-Rein-Straße 3, 37075 Göttingen, Germany

## Abstract

Superresolution fluorescence microscopy recently revealed a ~190 nm periodic cytoskeleton lattice consisting of actin, spectrin, and other proteins underneath the membrane of cultured hippocampal neurons. Whether the periodic cytoskeleton lattice is a structural feature of all neurons and how it is modified when axons are ensheathed by myelin forming glial cells is not known. Here, STED nanoscopy is used to demonstrate that this structure is a commonplace of virtually all neuron types *in vitro*. To check how the subcortical meshwork is modified during myelination, we studied sciatic nerve fibers from adult mice. Periodicity of both actin and spectrin was uncovered at the internodes, indicating no substantial differences between unmyelinated and myelinated axons. Remarkably, the actin/spectrin pattern was also detected in glial cells such as cultured oligodendrocyte precursor cells. Altogether our work shows that the periodic subcortical cytoskeletal meshwork is a fundamental characteristic of cells in the nervous system and is not a distinctive feature of neurons, as previously thought.

Actin and its binding partner spectrin are among the most abundant proteins in many eukaryotic cells and are key components of the subcortical cytoskeleton. Their importance in the nervous system is reflected by the fact that actin represents 3–5% of the brain’s total protein content, with spectrin amounting to ~3% of the total membrane proteins^1^,^2^,^3^. Recently, fluorescence nanoscopy and new labeling strategies enabled the discovery of a ~190 nm periodic organization of the actin and spectrin subcortical cytoskeleton in axons and a subset of dendrites of hippocampal neurons^4^,^5^,^6^,^7^.

Considering the high expression levels of both actin and spectrin throughout the nervous system, we speculated that the subcortical periodic organization might be a feature not only of excitatory hippocampal neurons, but also of other neuronal cell types and of non-neuronal cells. We therefore employed STimulated Emission Depletion (STED) nanoscopy of both fixed and living specimens. In this way we were able to identify a subcortical periodic lattice in the axons of other excitatory and inhibitory cultured neuronal cell types, and in highly specialized cells of the retina and, strikingly, in oligodendrocyte precursors. Finally, we analyzed actin organization at the nanoscale in intact nerve fibers, and found that actin and spectrin form a periodic structure even underneath the myelin coat. Our results suggest that this periodic organization is not an exclusive characteristic of hippocampal neurons but rather a general feature within the nervous system.

## Results

### Subcortical cytoskeletal periodicity is an axonal feature of all neuronal types

**Figure 1:**
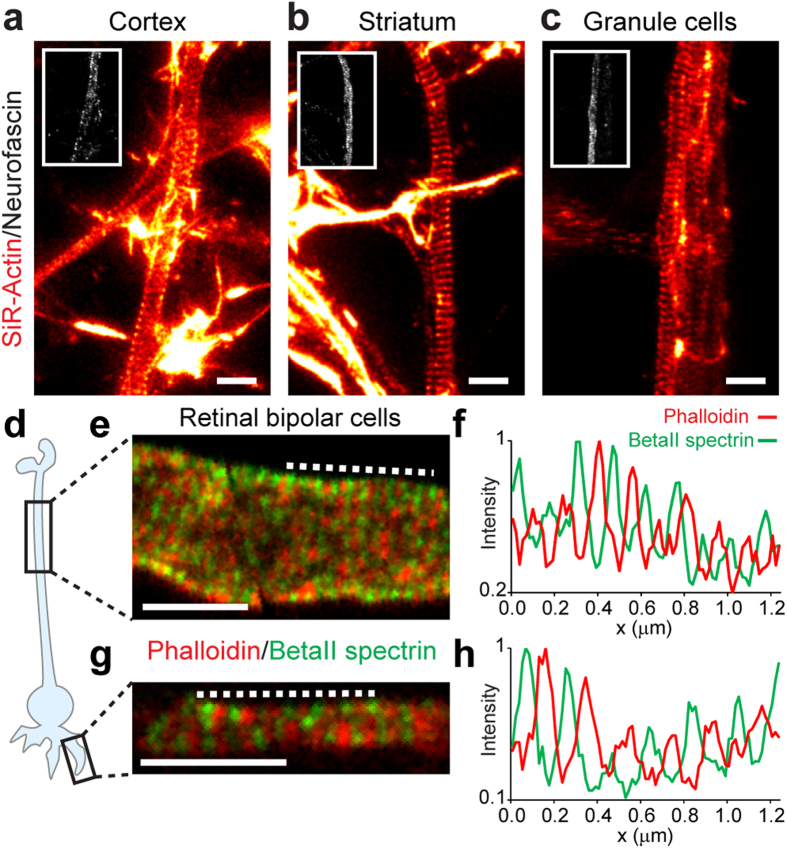
Subcortical cytoskeleton periodicity in the central nervous system. (**a–c**) Representative live STED images of cortical neurons (**a**), striatal neurons (**b**) and cerebellar granule cells (**c**) stained with SiR-Actin and neurofascin (insets). (**d**) Schematic drawing of a retinal bipolar cell with a long axon and short dendrites. (**e**,**g**) Two-color STED images of phalloidin- and betaII spectrin- stained retinal bipolar cell axon (**e**) and dendrite (**g**), highlighting the cytoskeletal periodic organization. (**f**) Line profile along the dashed line in (**e**). (**h**) Line profile along the dashed line in (**g**). All image data was smoothed. All scale bars: 1 μm.

All direct optical nanoscopy investigations into subcortical actin periodicity have so far been restricted to hippocampal neurons^4^,^5^,^6^,^7^,^8^. For this reason, we began by addressing the question whether this pattern is universal to different neuron types belonging to the central nervous system (CNS). To this end, we exploited SiR-Actin, a fluorescent reporter which labels endogenous actin in living cells with high specificity to subcortical actin, allowing exquisite detection sensitivity by imaging^6^,^7^. Since in hippocampal neurons the actin periodic pattern can be found only in a fraction of dendrites and its visualization strictly depends on the developmental stage of the cultures^6^, we focused our analysis on axons, where the lattice is clearly visible. Consequently, we used only cultures older than 5 days *in vitro* (DIV) and in which the axon initial segment (AIS) could be unequivocally identified by means of live-cell neurofascin staining^6^,^9^ (Fig. 1). Live STED imaging revealed the presence of a periodic actin pattern in the vast majority of the axons of cortical neurons, striatal neurons, and granule cells from the cerebellum (Fig. 1a–c respectively). Axons showed the presence of a long-range periodic subcortical actin lattice in 95.8% of cortical neurons (23 axons out of 24; spacing inferred from inter-peak separation of the fluorescence signals was 195 ± 25 nm ± s.d., n_peaks_ = 175; n_cells_ = 20), in 88% of striatal neurons (37 out of 42 axons; spacing 196 ± 28 nm, n_peaks_ = 181; n_cells_ = 21), and 90.6% of cerebellar granule cells (29 out of 32 axons; spacing 196 ± 23 nm, n_peaks_ = 182; n_cells_ = 21).

We then investigated the subcortical cytoskeleton of the highly specialized bipolar cells of the retina (Fig. 1d)^10^. We used dissociated, PFA-fixed mouse retinal bipolar cells^11^, which were stained against actin (using phalloidin) and betaII spectrin. Dual-color STED nanoscopy revealed that the subcortical cytoskeleton forms a periodic lattice all along the axon (31 cells out of 40 imaged, 77.5%), with betaII spectrin evenly expressed along the axonal shaft and intercalating actin (Fig. 1e,f). The spacing of the actin lattice was 169 ± 24 nm (n_peaks_ = 170; n_cells_ = 16). It is important to note that the periodicity of both actin and betaII spectrin is also present in the short dendrites (Fig. 1g,h), similarly to what has been reported in hippocampal neurons^5^,^6^.

**Figure 2:**
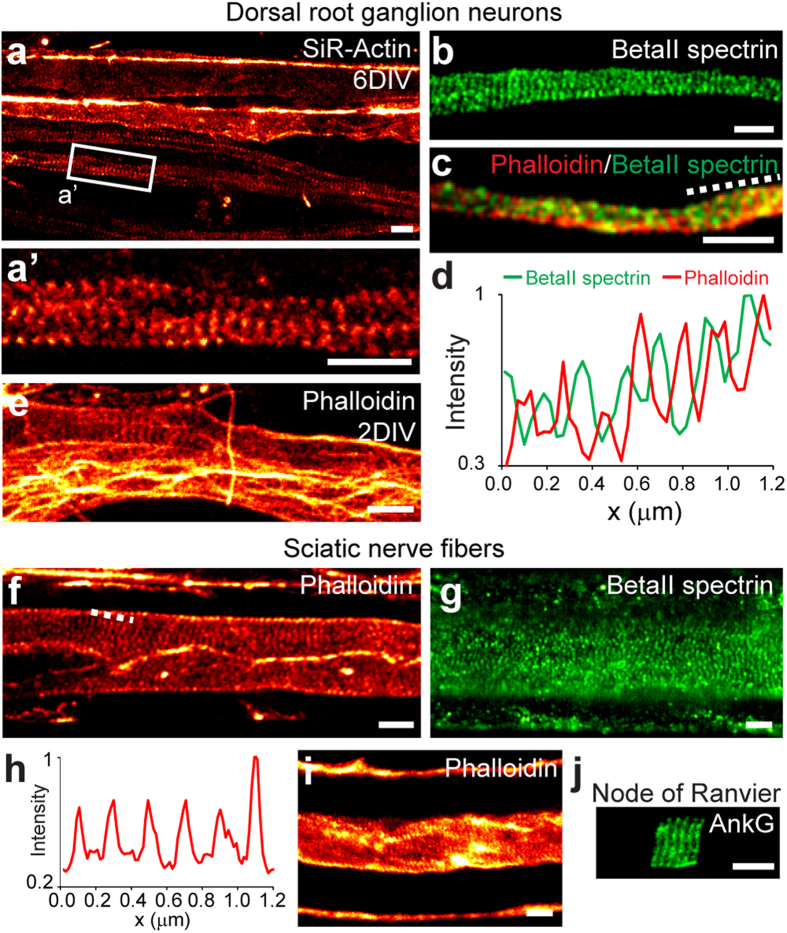
Subcortical cytoskeleton periodicity in the peripheral nervous system. (**a**) Living DRG neurons stained with SiR-Actin and imaged with STED nanoscopy at 6 DIV show long-range actin periodicity. (a’) Close-up of the area indicated in (**a**). (**b**) BetaII spectrin shows short-range periodic order in DRG neurons at 6 DIV. (**c**) BetaII spectrin and phalloidin co-staining shows an alternating pattern (6 DIV). (**d**) Line profile of phalloidin and betaII spectrin intensities along the dashed line in (**c**). (**e**) Phalloidin staining of fixed DRG shows the presence of the actin pattern already at 2 DIV. (**f**) Sciatic nerve stained with phalloidin and imaged with STED shows actin periodic organization at the internode. (**g**) BetaII spectrin periodic organization along the axon at internodal region. (**h**) Line profile of phalloidin intensity along the dashed line in (**f**). (**i**) STED image of another phalloidin stained internode highlights actin presence along the axon (periodic) and on the outermost myelin layer. (**j**) AnkyrinG staining at a node of Ranvier of a thin sliced sciatic nerve. All image data was smoothed. All scale bars: 1 μm.

Next, we moved to the peripheral nervous system (PNS), where a periodic organization of betaIV spectrin was already shown at the nodes of Ranvier^6^ (Fig. 2). We directly assessed the spatial organization of subcortical actin in the PNS by using primary cultured rat dorsal root ganglion (DRG) neurons at 2 to 8 DIV. Live STED nanoscopy of SiR-Actin-labelled cells revealed the presence of actin periodicity as early as 2 DIV, and by 6 DIV this became a prominent feature of every neuronal process (Fig. 2a,a’). The spacing of actin was 198 ± 26 nm (n_peaks_ = 140, n_cells_ = 14). Phalloidin staining of fixed DRG cells confirmed these results and, remarkably, allowed the identification of the actin periodicity already after 2 DIV (Fig. 2e, Supplementary Fig. 1a). In contrast, phalloidin had previously failed at labeling the subcortical actin lattice before 5 DIV in hippocampal neurons^4^,^5^,^6^. Immunostaining against betaII spectrin showed that it alternates with actin but its periodicity appears to be short-ranged, while SiR-Actin live-cell STED imaging experiments indicate a long-range actin order (Fig. 2b–d, Supplementary Fig. 1b).

Most of the axons in the central and peripheral nervous systems exhibit a myelin coat, and the cytoskeletal organization underneath is still an open question^12^. Therefore, we further investigated actin and betaII spectrin organization in myelinated sciatic nerve fibers from adult mice (>4 months old). As expected, actin is highly enriched at the nodes and paranodes, at the Schmidt-Lanterman incisures, and in the outermost myelin layer (see Supplementary Fig. 2a–d)^13^. F-actin is furthermore present at lower levels along the axon, while it is almost absent in compact myelin (Fig. 2f,i)^13^,^14^,^15^. BetaII spectrin localization follows a similar pattern, being present along the axon, with the exception of the nodes, and in the outermost myelin layer (see Supplementary Fig. 2e). Nanoscopy underneath the myelin coat turned out to be challenging because of the optical aberrations and scattering from numerous strata of lipid bilayers. Nevertheless, in experiments from 4 out of 6 animals we could detect actin periodic organization at the internodes (Fig. 2f,h,i, Supplementary Fig. 2a,c). BetaII spectrin also showed a periodic organization in this region, further confirming the presence of the subcortical lattice underneath the myelin coat (Fig. 2g). The actin inter-peak spacing in sciatic nerves was 186 ± 23 nm (n_peaks_ = 281, n_axons_ = 28). It should be mentioned, however, that the variability of spacings between different animals was high, ranging from 177 ± 22 nm to 198 ± 18 nm (see Supplementary Fig. 3). The differences may be ascribed to the technical difficulties of the sample preparation and mounting for nanoscopy, to the complex three-dimensional organization of the specimens, and to the imaging through compact myelin sheets. Due to very high actin concentrations at the nodes and paranodes, it was not possible to identify the fine subcortical actin structure in these compartments (see Supplementary Fig. 2b). However, STED imaging of thin sections (250–300 nm thin) demonstrated that also the nodal-resident protein AnkyrinG (a scaffolding protein that links the spectrin-actin subcortical cytoskeleton to the membrane^16^) is periodically organized at the nodes of Ranvier (Fig. 2j), as it is in the AIS of cultured hippocampal neurons^5^ and as has been shown for betaIV spectrin^6^.

We conclude that the periodic subcortical cytoskeleton pattern is present in virtually all the axons of both the CNS and the PNS, even below the myelin sheet.

### Oligodendrocyte precursors also exhibit an actin/spectrin pattern

**Figure 3:**
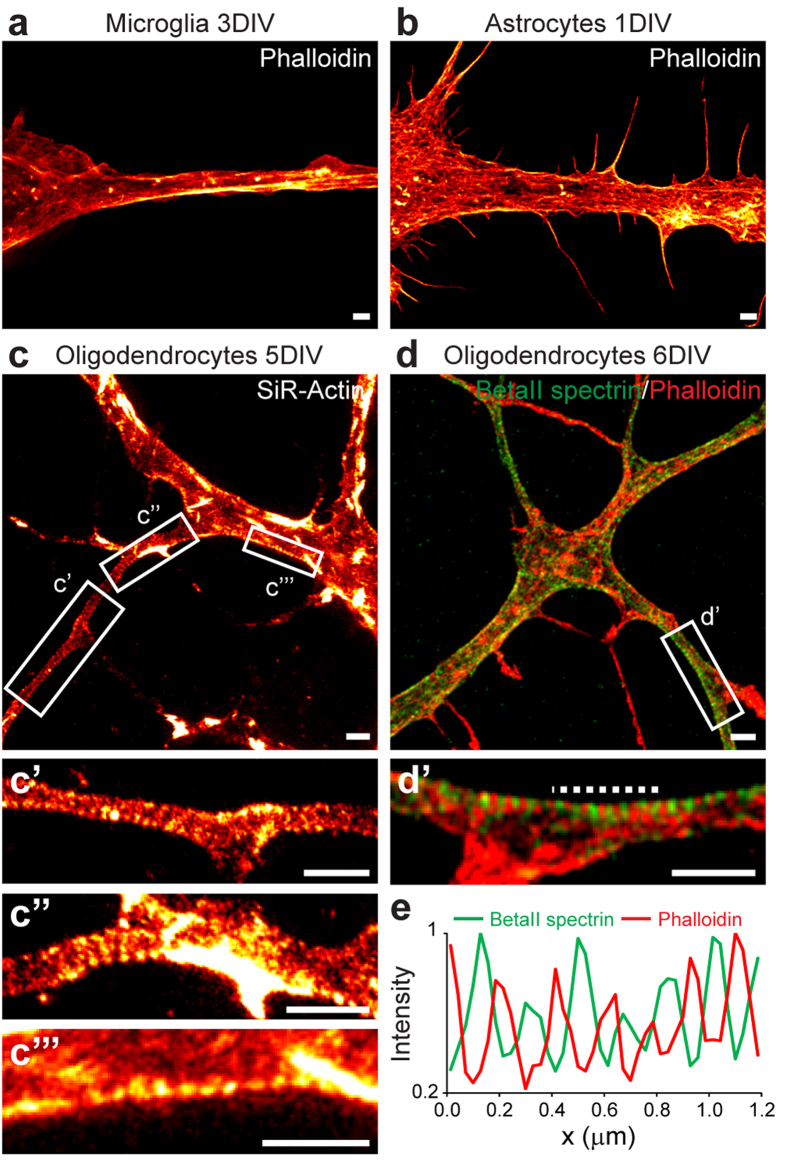
The subcortical cytoskeleton in glial cells. (**a**,**b**) Representative STED images of phalloidin stained microglial cells at 3 DIV (**a**) and astrocytes at 1 DIV (**b**). (**c**) Living oligodendrocytes stained with SiR-Actin and imaged with STED nanoscopy at 5 DIV. (c’,c’’,c’’’) Close-ups of the areas indicated in (**c**). (**d**) BetaII spectrin and phalloidin co-staining in oligodendrocytes at 6 DIV. (d’) Close-up of the area indicated in (**d**) shows an alternating pattern of actin and betaII spectrin. (**e**) Line profile along the dashed line in (d’). All image data was smoothed. All scale bars: 1 μm.

The fact that spectrin is expressed also in glial cells^17^,^18^,^19^ raised the question of the existence of the subcortical cytoskeletal organization in these cells, whose morphology can feature elongated structures. The main glial cell types are astrocytes, microglial cells and oligodendrocytes. Unfortunately, microglial cells could not be successfully stained with SiR-Actin and therefore were imaged after PFA-fixation and phalloidin staining. Astrocytes and oligodendrocytes, on the other hand, could be imaged live, with SiR-Actin staining (Fig. 3). Even when focusing on thin and long processes of microglial cells (at 1 to 7 DIV) and astrocytes (living or fixed, at 1 to 5 DIV), we were not able to detect any obvious subcortical actin/spectrin pattern (Fig. 3a,b, Supplementary Fig. 4). In contrast, in differentiating oligodendrocytes, which have dendritic-like processes, SiR-Actin highlighted the presence of short-range actin periodicity with spacings of 190 ± 28 nm (n_peaks_ = 132, n_cells_ = 18) (Fig. 3c,c’’’). Phalloidin staining of fixed oligodendrocytes confirmed this finding, and, in combination with betaII spectrin immunostaining, showed the typical alternating pattern (Fig. 3d,e). Therefore, these data demonstrate that the subcortical cytoskeletal periodicity of actin and spectrin is not a unique feature of neurons.

## Discussion

Far-field optical nanoscopy allows the visualization of previously unseen features of the cytoskeleton, among them the existence of a subcortical ~190 nm periodic structure found in the neurites of hippocampal neurons^4^,^5^,^6^,^7^. This structural architecture is reminiscent of the cytoskeletal organization found in red blood cells^20^,^21^ and the cytoskeletal lattice that has been described in the motoneuron axon of *Drosophila*^22^.

**Figure 4:**
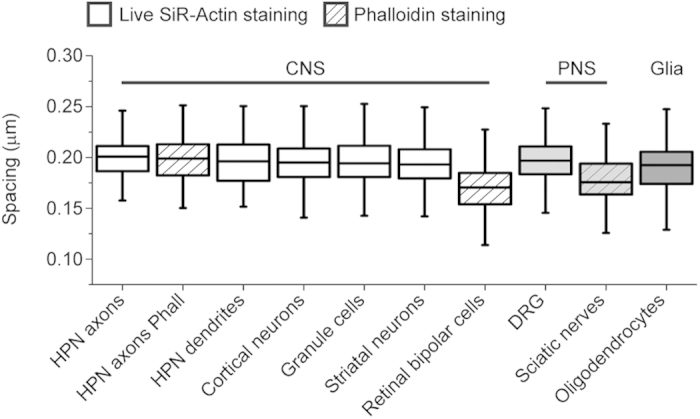
Comparison of actin spacing in the different neuronal cell types. Box plot of the actin inter-peak spacing in the different cells. All measurements have been performed on the axons (when applicable). HPN: hippocampal neurons. Data for HPN axons, HPN axon phalloidin, and HPN dendrites taken from the experiments performed in^6^ (n_peaks_ = 65, n_cells_ = 7 for HPN axons; n_peaks_ = 80, n_cells_ = 5 for HPN axons Phall; n_peaks_ = 50, n_cells_ = 4 for HPN Dendrites). Data for the other cell types and tissues are reported in the text.

Here, we have expanded this finding to other neuronal types of the CNS and the PNS, demonstrating the presence of a periodic pattern even in highly specialized cells such as retinal bipolar cells, and, for the first time, underneath the myelin coat. Notably, this very same feature of the subcortical cytoskeleton was also found in oligodendrocytes, which are the cells responsible for the myelination of axons in the CNS^23^. The actin periodicity is analogous in the axons of both excitatory neurons (cortical neurons, granule cells, DRGs) and inhibitory neurons (striatal neurons are mainly GABAergic^24^), and is similar to the one found in hippocampal axons and dendrites (Fig. 4). However, in retinal bipolar cells (which are not myelinated) the spacing was found to be slightly shorter. This distinct difference cannot be ascribed to the spectrin isoform (as betaII spectrin intercalates with actin), nor to the PFA fixation (Fig. 4, compare hippocampal neurons (HPN) stained with SiR-Actin or phalloidin), nor to the lack of myelination (most of the experiments have been performed on unmyelinated cultured neurons). Further investigations are therefore obviously required to confirm this result and understand the possible function and fine tuning of the lattice spacing.

With regard to the PNS, we have provided direct evidence of actin and betaII spectrin periodicity all along the neurites of unmyelinated DRG cultures, similarly to what has been reported for hippocampal neurons^5^. A difference to the previous studies^4^,^5^,^6^ is that phalloidin in our hands was capable of detecting the actin periodicity as early as 2 DIV in the DRG cells, while in hippocampal neurons its periodicity could first be unequivocally observed with phalloidin at 5 DIV (even if SiR-Actin live imaging detected it earlier). This varying phalloidin sensitivity may be due to slightly different assembly mechanisms of the actin ultrastructure or to the presence of stabilizing proteins.

Imaging of the periodic actin and spectrin lattice in sciatic nerve fibers provides an answer to one of the questions that was raised when the periodic pattern was first described: how is the cytoskeleton organized underneath the myelin coat^12^? Our data show that there is no substantial difference in the organization of the subcortical cytoskeleton between unmyelinated and myelinated axons, since a periodic lattice is found in both cases. Subcortical organization at the nodes of Ranvier of actin itself remains elusive, even if it was possible to demonstrate in this work that another nodal marker beside betaIV spectrin^6^, namely AnkyrinG, organizes with clear periodicity.

The absence of an actin/spectrin periodicity in microglial cells and astrocytes could be explained in two ways, one biological and one technical. These cell types are quite dynamic and the subcortical cytoskeletal lattice might be assembled only in stable processes. Moreover, when grown in culture these cells have a two-dimensional morphology that is quite different from the one they exhibit under physiological conditions, where they contact and sustain synaptic structures in a three-dimensional environment^25^. With this in mind, analysis of glial cells *in vivo* could lead to different results. Moreover, the actin lattice appears as a dim structure and requires extremely low background to be appreciated. We cannot therefore rule out that the presence of high actin content in the cytosol of glial cells prevented the identification of the faint actin periodicity. On the other hand, oligodendrocyte precursors display the presence of the actin/spectrin lattice. However, F-actin disassembly is a key process in the later myelin enwrapping phase, and, at the same time, both F-actin and betaII spectrin become virtually absent from compact myelin^14^,^15^. These cytoskeletal differences during the myelination process suggest the presence of control mechanisms for the actin/spectrin lattice formation and disassembly.

In conclusion, in this work we have demonstrated that the subcortical periodicity of the cytoskeleton is a much more general feature of the nervous system, being present in all tested neuron types, in myelinated axons, and even in oligodendrocyte precursors. Impairments in the expression of spectrins can be embryonically lethal in the case of knockout animals^26^,^27^ or lead to severe diseases in humans (reviewed by^28^,^29^,^30^), including progressive neurodegenerative disorders and cerebral hypomyelination^31^,^32^. Therefore, it would be of high interest to explore how these deficiencies affect the subcortical lattice organization both in neurons and in oligodendrocytes, in particular during the myelination. In this respect, optical nanoscopy will play a central role and hopefully will allow to directly connect the ultrastructural organization of the cytoskeleton with mutations and disease.

## Materials and Methods

### Neuronal cell culture

Cultures of cortical neurons were prepared from Wistar rats of mixed sex at postnatal day P0–P1 in accordance with Animal Welfare Law of the Federal Republic of Germany (Tierschutzgesetz der Bundesrepublik Deutschland, TierSchG) and the Regulation about animals used in experiments (1^st^ August 2013, Tierschutzversuchsverordnung). For the procedure of sacrificing rodents for subsequent preparation of any tissue, all regulations give in §4 TierSchG are followed. Since sacrificing of animals is not an experiment on animals according to §7 Abs. 2 Satz 3 TierSchG, no specific authorization or notification is required. The preparation followed the same procedure as for hippocampal neurons described in^6^.

Rat brain striatal neurons, rat cerebellar neurons, and rat postnatal dorsal root ganglion neurons were purchased from Lonza (cat. R-Cp-502, R-CB-503, and R-DRG-505 EA, respectively) and cultured following the manufacturer’s instructions. All cells were maintained in a humidified 5% CO_2_ incubator at 37 °C.

Mouse retinal bipolar cells were prepared as described previously^33^ and were a kind gift of Prof. David Zenisek (Yale University School of Medicine).

### Glial cell preparation

Primary cell cultures were prepared as described previously^34^. Briefly, brains were extracted from P1 NMRI mice. After trypsinization for 10 min, cells were mechanically dissociated and seeded into cell culture flasks. Primary glial mixed cultures were grown in Basal Medium Eagle supplemented with 10% horse serum, 1% GlutaMax, and 1% pen/strep. After 7–8 days, primary oligodendrocyte progenitor cells (OPCs) were manually shaken off the mixed glial culture and plated onto poly-L-lysine (PLL) coated coverslips. OPCs were kept in a humidified 37 °C incubator, supplemented with 7.5% CO_2_ in Super SATO differentiation medium. After shaking off the OPCs, fresh DMEM growth medium (supplemented with 10% fetal calf serum (FCS), 1% GlutaMax, and 1% pen/strep) was added to the remaining astrocytes and kept for 24–72 hours to allow their recovery. Astrocytes were harvested by trypsinization, seeded onto PLL-coated coverslips, and cultured in DMEM (all media and reagents from Gibco).

The preparation of microglia was performed as described previously^35^. Glial mixed cultures were treated with 10% microglia colony stimulating factor produced by L919 fibroblasts. After 3–5 days, microglia were harvested by manual shaking and seeded onto PLL-coated coverslips in DMEM growth medium.

### Reagents

SiR-Actin was a kind gift of Prof. Kai Johnsson and Dr. Grazvydas Lukinavicius (EPFL, Lausanne, Switzerland) and is now commercially available (Spirochrome, cat. SC001).

The antibodies used in this study are: anti-betaII spectrin (BD Biosciences, cat. 612563, 1:200 dilution); anti-neurofascin (NeuroMab, cat. 75–172; 1:200 dilution); anti-AnkyrinG (Santa Cruz Biotechnologies, cat. sc-12719; 1:200 dilution); anti-mouse secondary antibody (Dianova, cat. 515-005-0039) was custom-labeled with the dyes STAR580 (Abberior, cat. 1-0101-005) or STAR635 (Abberior, cat. 2-0205-002). Phalloidin was coupled to STAR635 (Abberior, cat. 2-0205-002-5, 1:100 dilution).

### SiR-Actin live staining

For live cell imaging, cells were stained with 100 nM SiR-Actin in their growth media in a humidified 5% CO_2_ incubator at 37 °C for 30–60 min. After quickly washing, cells were imaged in artificial cerebrospinal fluid (ACSF buffer).

### Immunostaining

For immunostainings, cells were washed with PBS and fixed in 4% PFA in PBS (pH 7.4) for 20 min at room temperature, quenched with ammonium chloride and glycine (100 mM each) for 5 min, permeabilized with 0.1% Triton X-100 for another 5 min and blocked in PBS supplemented with 1% BSA for 30 min. Both primary and secondary antibody, and phalloidin incubations were performed in PBS for 1 hour at room temperature or overnight at 4 °C. Samples were mounted in Mowiol supplemented with DABCO.

### Sciatic nerves preparation

Sciatic nerves were extracted from mice at least 4 months old both C57BL/6 and B6N (mixed sexes) and fixed in 4% PFA. Free-floating fibers were then partially teased, permeabilized with 0.5% Triton X-100 in PBS for 45 min, or with ice cold methanol for 20 min in the case of betaII spectrin staining. Nerves were then blocked in PBS containing 1% BSA for 45 min. Both primary and secondary antibody incubations were performed for 1h at RT or overnight at 4 °C in PBS supplemented with 0.05% Triton X-100. After each step, samples were rinsed 3 times for 10 min with 0.05% Triton X-100. At the end, samples were teased on a coverslip and mounted in Mowiol supplemented with DABCO.

For the preparation of thin sections, stained samples were embedded in melamine, and processed as previously described^6^.

### Imaging

Imaging was performed on a home-built two-color STED nanoscope^36^ or on a two-color Abberior STED 775 QUAD Scanning microscope (Abberior Instruments GmbH, Göttingen, Germany) equipped with 561 nm, 594 nm, and 640 nm pulsed excitation lasers, a pulsed 775 nm STED laser, and a 100x oil immersion objective lens (NA 1.4).

### Image analysis

All acquired images were processed and visualized using the ImSpector software package (Max-Planck Innovation) and ImageJ (imagej.nih.gov/ij/). Smoothing was performed using a low-pass Gaussian filter with the ImSpector software. Brightness and contrast were applied uniformly to all portions of the image. Line profiles were measured with ImageJ along a 3–5 pixel wide line. Inter-peak distances were determined using the multi-peak fitting function in OriginPro8.5.

## Additional Information

**How to cite this article**: D’Este, E. *et al.* Subcortical cytoskeleton periodicity throughout the nervous system. *Sci. Rep.* **6**, 22741; doi: 10.1038/srep22741 (2016).

## Supplementary Material

Supplementary Information
