# Supplementary material for: Subcortical cytoskeleton periodicity throughout the nervous system
Source: Sci Rep. 2016 Mar 7;6:22741. doi: 10.1038/srep22741 (PMC4779989; doi:10.1038/srep22741)
Supplement: Supplementary Information [file srep22741-s1.pdf]

Supplementary information

Subcortical cytoskeleton periodicity throughout the nervous system

Elisa D'Este[1, #, *], Dirk Kamin[1, #], Caroline Velte[2], Fabian Göttfert[1], Mikael Simons[2], and Stefan W. Hell[1, *]

[#] Contributed equally to this work.

[1] Department of NanoBiophotonics, Max Planck Institute for Biophysical Chemistry, Am Fassberg 11, 37077 Göttingen, Germany

[2] Department of Cellular Neuroscience, Max Planck Institute for Experimental Medicine, Hermann-Rein-Straße 3, 37075 Göttingen, Germany

[*] Correspondence: edeste@mpibpc.mpg.de; shell@mpibpc.mpg.de. Department of NanoBiophotonics, Max Planck Institute for Biophysical Chemistry, Am Fassberg 11, 37077 Göttingen, Germany

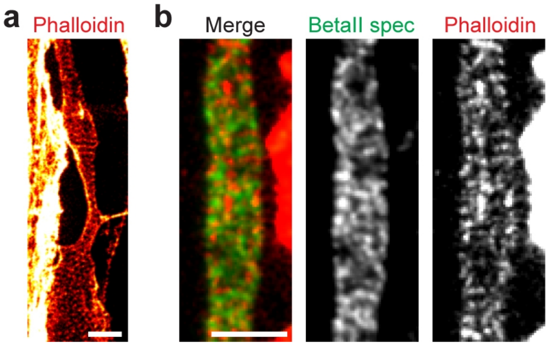

Supplementary Figure 1. Actin and spectrin organization in DRG cells at 6 DIV. (**a**) Phalloidin staining of DRG cells confirms the presence of the actin periodicity in this neuron type. It is important to mention that while with SiR-Actin live-labeling all the axons exhibit the periodic lattice, this feature is not as prominent using phalloidin staining. (**b**) BetaII spectrin (green) and phalloidin (red) co-staining of an axon and corresponding single channel images. All image data was smoothed. All scale bars: 1 µm.

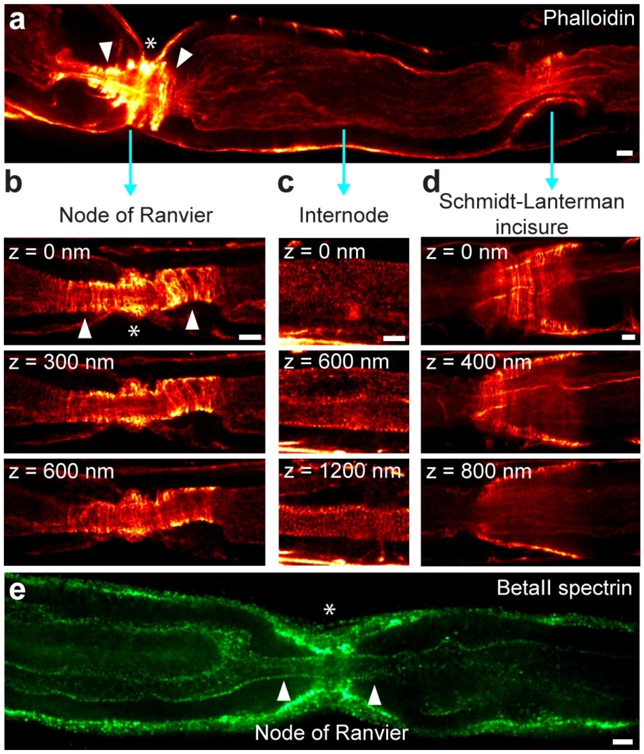

Supplementary Figure 2. Actin and spectrin organization at the nanoscale in sciatic nerve fibers. (**a**) Sciatic nerve stained against actin using phalloidin and imaged with STED shows high actin levels at the node of Ranvier (asterisk indicates the node, arrowheads point to the paranodes), at the Schmidt-Lanterman incisure, and on the outermost myelin layer. Actin is also present at lower levels at the internode. (**b-d**) Examples of optical sections of actin organization at a node of Ranvier (b, asterisk indicates the node, arrowheads point to the paranodes), at the internode (c), and at a Schmidt-Lanterman incisure (d), from different samples. (**e**) STED image of a sciatic nerve in which betaII spectrin is present at the internodes, paranodes, and on the outermost myelin layer (asterisk indicates the node, arrowheads point to the paranodes). All image data was smoothed. All scale bars: 1 µm.

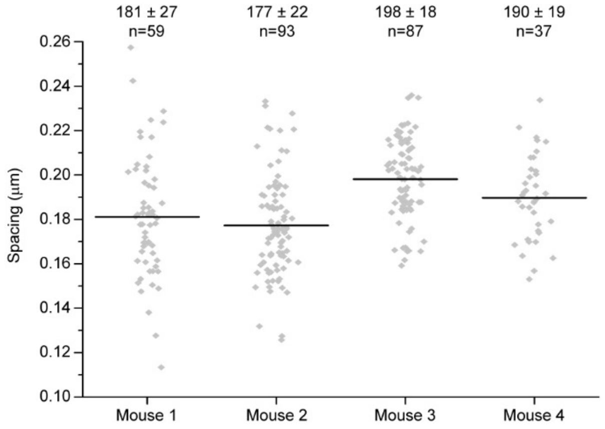

Supplementary Figure 3. Actin inter-peak spacing in different mice. The plot shows the spacings measured for each animal. Black bars indicate the average values, reported on top of the datasets ± standard deviation. n indicates the number of spacings measured.

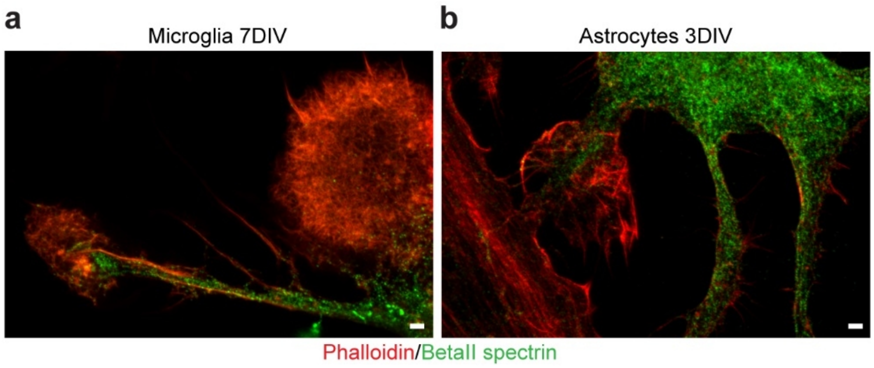

Supplementary Figure 4. Actin and betaII spectrin organization at the nanoscale in (**a**) microglial cells at 7 DIV and (**b**) astrocytes at 3 DIV. No obvious periodicity is visible. All image data was smoothed. Scale bars: 1 µm.
